# Clinical Validation of the Somatic FANCD2 Mutation (c.2022-5C>T) as a Novel Molecular Biomarker for Early Disease Progression in Chronic Myeloid Leukemia: A Case–Control Study

**DOI:** 10.3390/hematolrep16030045

**Published:** 2024-07-08

**Authors:** Nawaf Alanazi, Abdulaziz Siyal, Sulman Basit, Masood Shammas, Sarah Al-Mukhaylid, Aamer Aleem, Amer Mahmood, Zafar Iqbal

**Affiliations:** 1 Division of Hematology/Oncology, Department of Pediatrics, King Abdulaziz Hospital, College of Applied Medical Sciences (CoAMS), King Saud Bin Abdulaziz University for Health Sciences, Al-Ahsa 36428, Saudi Arabia; anazinm@ngha.med.sa; 2 Stem Cell Unit, Department of Anatomy, College of Medicine, King Saud University, Riyadh 11495, Saudi Arabia; 3 Centre for Genetics and Inherited Diseases, Taiba University, Madinah 42353, Saudi Arabia; sakbar@taibahu.edu.sa; 4 Dana Farbar Cancer Institute, University of Harvard, Boston, MA 02138, USA; masood_shammas@dfci.harvard.edu; 5 Clinical Laboratory Department, Johns Hopkins Aramco HealthCare (JHAH), Alahsa 36423, Saudi Arabia; almukhaylid034@ksau-hs.edu.sa; 6 Alumni, GEM, CLSP, CoAMS-A, KSAU-HS, Al-Ahsa 36428, Saudi Arabia; 7 Department of Medicine, Division of Hematology/Oncology, College of Medicine, King Khalid University Hospital, King Saud University, Riyadh 11472, Saudi Arabia; ameralem@ksu.edu.sa; 8 Genomic & Experimental Medicine Group (GEM) Molecular Oncology/Hematology Group (MOH) & Quality Assurance and Accreditation Unit (QAAA), & Clinical Laboratory Sciences Program (CLSP), College of Applied Medical Sciences (CoAMS-A), King Abdullah International Medical Research Centre (KAIMRC), King Saud Bin Abdulaziz University for Health Sciences (KSAU-HS), Saudi Society for Blood and Marrow Transplantation (SSBMT), King Abdulaziz Medical City, National Guard Health Affairs, Al-Ahsa 31982, Saudi Arabia; 9 Pakistan Society for Molecular and Clinical Hematology, Lahore 54000, Pakistan; 10 Hematology, Oncology & Pharmacogenetic Engineering Sciences Group (HOPES), Division of Next-Generation Medical Biotechnology (NeMB), Department of Biotechnology, Qarshi University, Lahore 54000, Pakistan; 11 Hematology, Oncology & Pharmacogenetic Engineering Sciences Group (HOPES), Centre for Applied Molecular Biology (CAMB), University of the Punjab, Lahore 54590, Pakistan

**Keywords:** chronic myeloid leukemia, CML progression, poor survival, genomic instability, DNA repair, FANCD2

## Abstract

**Background:** Chronic myeloid leukemia (CML) results from chromosomal translocation t(9;22) leading to the formation of the BCR-ABL fusion oncogene. CML has three stages: the chronic phase (CP), the accelerated phase (AP), and the blast crisis (BC). Tyrosine kinase inhibitors (TKIs) have revolutionized the treatment of CML. TKIs work well in CP-CML, and these patients have a survival rate similar to the normal population, but TKIs are less effective in advanced-phase CML. Even with current advances in treatment, BC-CML patients have an average overall survival of less than a year. Early recognition of CML patients at risk of disease progression can help in timely interventions with appropriate TKIs or other therapeutic modalities. Although some markers of disease progression like BCR-ABL kinase domain, ASXL1, and GATA2 mutations are available, no universal and exclusively specific molecular biomarkers exist to early diagnose CML patients at risk of CML progression for timely therapeutic interventions to delay or minimize blast crisis transformation in CML. A recent study found that all BC-CML patients harbored the FANCD2 (c.2022-5C>T) mutation. Therefore, the current study was designed to detect this FANCD2 mutant in AP-CML (early progression phase) and to clinically validate its potential as a novel molecular biomarker of early CML progression from CP to AP. **Methods:** Our study comprised 123 CP-CML (control group) and 60 AP-CML patients (experimental group) from 2 oncology centers, from January 2020 to July 2023. Mean hemoglobin level, WBC count, platelet count, treatment type, hepatomegaly, splenomegaly, and survival status of AP-CML patients were significantly different from those of CP-CML patients. However, as these clinical parameters cannot help in the early detection of patients at risk of CML progression, there was a need for a clinically validated biomarker of AP-CML. DNA was extracted from the patients’ blood samples, and the FANCD2 gene was sequenced using an Illumina NextSeq500 next-generation sequencer (NGS). **Results:** The NGS analysis revealed a unique splice-site mutation in the FANCD2 gene (c.2022-5C>T). This mutation was detected in the majority (98.3%) of AP-CML patients but in none of the CP-CML patients or healthy control sequences from genomic databases. The mutation was confirmed by Sanger sequencing. FANCD2 is a member of the Fanconi anemia pathway genes involved in DNA repair and genomic stability, and aberrations of this gene are associated with many cancers. **Conclusions:** In conclusion, our study shows that the somatic FANCD2 (c.2022-5C>T) mutation is a new molecular biomarker for early CML progression. We recommend further clinical validation of this biomarker in prospective clinical trials.

## 1. Introduction

Chronic myeloid leukemia (CML) is a chronic myeloproliferative malignancy of stem cells that is manifested in blood and bone marrow [1]. It is caused by a reciprocal translocation between chromosome 9 and 22 [t(9; 22)], leading to the formation of Philadelphia (Ph) chromosome [2]. This translocation results in BCR-ABL fusion oncogene responsible for the onset of malignant proliferation in the myeloid lineage of hematopoietic stem cells [3]. Estimates of the annual prevalence of CML range from 0.6 to 2.0 cases per 100,000 population, or around 10–15% of newly diagnosed adult cases of leukemia [1,4]. The median age at diagnosis of CML is between 57 and 60 years, and it is more common in males, for whom the prevalence of this disease is 1.2–1.7% higher [5,6]

Tyrosine kinase inhibitors (TKIs) have revolutionized CML treatment; however, there are still challenges in the management of patients with advanced-phase CML [7]. CML has three disease phases the chronic phase (CP), the accelerated phase (AP), and the blast crisis (BC) [8]. TKIs are very effective in CP-CML, improving the overall survival rate from 20% to more than 90% [7,9]. This has led to an overall survival of CP-CML patients similar to the general population, at least in technologically advanced countries [10]. Nevertheless, patients in the early progression phase (AP-CML) and advanced progression phase (BC-CML) show resistance to TKIs [11]. Despite all advancements in the treatment modalities, the average overall survival of BC-CML patients is less than a year, with limited options to treat these patients [12]. Early recognition of CML patients at risk of disease progression can help to delay or even avoid CML progression by timely interventions with third- and/or fourth-generation TKIs [13]. Although some markers of disease progression like BCR-ABL kinase domain mutations and some non-BCR-ABL gene mutations like ASXL1, GATA2, etc., have been reported, no exclusively specific and universal molecular biomarkers exist for the timely detection of CML patients at risk of disease progression [14,15]

There are several mechanisms involved in the initiation and progression of different types of cancers, including DNA repair defects leading to genomic instability [16]. Fanconi anemia (FA) is a rare autosomal recessive disorder characterized by gene mutations that are predominantly involved in DNA damage response or repair [17]. The FANC genes play a crucial role in the FA pathway, regulating DNA damage responses through complicated processes like ubiquitination, phosphorylation, and degradation signals, all of which are required for genome stability and genomic integrity [18]. Due to increased genomic instability, the FANC gene dysfunction increases the chances of developing various hematological and solid malignancies [19]. In a recent study, the FANCD2 mutation was found to be associated with terminal CML progression [20]. The current study was designed to find out the potential and clinical validation of mutated FANCD2 as a biomarker of early CML progression in AP-CML using a case-control study design.

## 2. Materials and Methods

Patient selection and recruitment: This study was conducted on CML patients enrolled at King Abdulaziz National Guard Hospital, Al-Ahsa, Saudi Arabia, and Hayat Abad Medical Center (HMC), Peshawar, from January 2020 to July 2023. A total of 183 CML patients were included in this study. The experimental group comprised 60 AP-CML patients, while 123 age/gender-matched CP-CML patients served as controls. All patients were initially treated with imatinib mesylate (IM), and patients with IM resistance received nilotinib (NI). The European Leukemia Net guidelines 2020 were followed to determine the criteria for CP and AP diagnosis and treatment responses [21,22]. Standard terminologies version 4.03 was used to classify the hematological and other adverse events [23].

The regulations of the Declaration of Helsinki were followed throughout the study. All patients included in the study provided written informed consent [24,25]. The approval of study protocols was obtained from the King Abdullah International Medical Research Center (KAIMRC); King Saud bin Abdulaziz University for Health Sciences (KSAU-HS), Saudi Arabia; and Hayat Abad Medical Center (HMC), Peshawar, Pakistan.

### 2.1. Sample Collection and DNA Extraction

Peripheral blood samples were collected in 3–5 mL EDTA tubes (BD Vacutainer Systems, Franklin Lakes, NJ, USA) from all age groups and clinical phases of CML patients and stored at −70 °C for further examination. Venous blood samples were obtained from registered CML patients 2–3 times monthly, for follow-up and medication refills, during their visits to the outpatient departments (OPD) of the Hematology Department, King Abdulaziz National Guard Hospital, Al-Ahsa, Saudi Arabia, and Hayat Abad Medical Center, Peshawar. All blood samples, DNA extraction kits, and reagents were set to room temperature (15–25 °C) before DNA extraction by using a 56 °C water bath. Genomic DNA extraction from blood samples was performed using the Qiamp DNA Extraction Kit (Qiagen, Valencia, CA).

### 2.2. Sequencing of FANCD2 Using Next-Generation Sequencing (NGS)

To represent each clinical phase of the disease (CP and AP), CML patient samples were selected and processed for NGS (Gnirke, 2009). An Illumina^®^ DNA Prep with Enrichment, (S) Augmentation kit (Cat. # 20025523) was utilized for target enrichment [26,27]. The first step of NGS was DNA fragmentation, followed by tagmentation. Afterward, tagmented DNA fragments were amplified and then purified using magnetic beads. Next, Oligos were utilized to capture target regions. Enriched libraries were amplified by PCR and quantified using a Qubit fluorometer, while an Agilent Bioanalyzer (Agilent Technologies, Santa Clara, CA, USA) was equipped to measure the library size distribution. Finally, cluster generation and exome sequencing were performed using the Illumina NextSeq500 instrument (Illumina Inc., San Diego, CA, USA) by loading the quantified DNA libraries on the flow cell [20,27].

### 2.3. Next-Generation Sequencing (NGS) Data Analysis

The conversion of output files, BCL files to FASTQ files, was performed by BCL2FASTQ software Version 2.20. Alignment of the FASTQ files to the human genome was performed by BWA Aligner, applying the BWA-MEM algorithm. Variants were called by the Genome analysis tool kit (GATK) (Illumina Inc., San Diego, CA 92122, USA). Illumina Variant Studio (Illumina Inc., San Diego, CA 92122, USA) was used for the annotation and filtration of genomic variants [27].

Strategy for screening of common biomarker for AP-CML: Although some markers of disease progression like BCR-ABL kinase domain, ASXL1, and GATA2 mutations are available, no universal and exclusively specific molecular biomarkers exist to early diagnose CML patients at risk of CML progression for timely therapeutic interventions to delay or minimize blast crisis transformation in CML (BC-CML). In our previous study, our group found that all BC-CML patients harbored the FANCD2 mutant 2022-5C>T [20]. During WES-based screening, they shortlisted only those genes that were mutated in all BC-CML patients and none of the CP-CML patients or healthy controls, as their objective was to find a “Common and very specific biomarker for BC-CML” (Absar et al., 2020, https://pubmed.ncbi.nlm.nih.gov/33361032/, accessed on 1 January 2024). We utilized the same approach to shortlist that gene(s) mutated in all or a majority (90% or more) of AP-CML or BC-CML patients. Moreover, previous study needed to be reproduced, and further clinical validation of that study using a larger number of CML patients was required. Our NGS in the current study found that the same FANCD2 gene mutated in all but one (59/80 = 98.3%) of the AP-CML patients. Therefore, the FANCD2 gene was screened for further clinical validation studies.

### 2.4. Primary Analysis

The FANCD2 gene was analyzed in all CML patients to detect shared biomarkers of CML progression. Filtration strategies that relied on calling rare variants and excluding intron and synonymous variants were applied to modify the Excel file presenting the NGS. Furthermore, all variants with known prediction were removed, either benign (B) or tolerant (T). Some variants were considered as B when they had 70% or more of B, while other variants were classified as T when T’s frequency was 70% or more [28]. Variants with more than 0.005 population frequency in the dbSNP and Exome Sequencing Project (ESP) database were also eliminated. Thus, variant calling was only limited to variants with intermediate and high protein effects along with splice variants, resulting in about 124 rare variants. Moreover, data were further analyzed to investigate novel gene mutations that are present in AP-CML patients but not in CP-CML patients and healthy controls, suggesting its role in disease progression [29,30]. Access to data created by next-generation sequencing can be obtained from NCBI, to which the data were submitted, at https://www.ncbi.nlm.nih.gov/bioproject/PRJNA1119181, accessed on 3 March 2024). Supplementary Data related to the NGS of FANCD2 were submitted to an online data server (Supplementary Table S1; https://doi.org/10.6084/m9.figshare.25957204.v2, accessed on 3 March 2024).

### 2.5. Validation of Mutation by Sanger Sequencing

Samples were prepared using an ABI Prism 3730 Genetic Analyzer (Applied Biosystems, CA, USA) and ABI PRISM Big Dye Terminator Cycle Sequencing Ready Reaction kit (Applied Biosystems, CA, USA) and Amplification of samples was performed via PCR. Variants identified through NGS were validated by Sanger sequencing [31]. Forward and reverse sequencing of PCR-amplified FANCD2 fragments were performed by the Sanger sequencer and mutational analysis was carried out as described earlier [20].

Analysis of the FANCD2, c. 2022-5C>T: Genomic mapping of FANCD2, c. 2022-5C>T was carried out using NCBI Variation Viewer (https://www.ncbi.nlm.nih.gov/variation/view/?assm=GCF_000001405.25, accessed on 3 March 2024. GnomAD browser was utilized to confirm the splice-site location of the mutant and its frequency in the general population (https://gnomad.broadinstitute.org/variant/3-10106408-C-T?dataset=gnomad_r2_1, accessed on 3 March 2024. The sequence data file generated from VCF files was utilized to see the VAF of FANCD2, c. 2022-5C>T.

### 2.6. Statistical Analysis of Patient Clinical Data

Categorical variables were represented with percentages and absolute numbers, while continuous variables were measured with mean and median according to the normality test. Chi-Square and Fisher’s exact test were utilized to compare categorical data of two groups, depending on applicability, while the comparison of two groups of continuous data was conducted by a two-sample independent test or Mann–Whitney U test, depending on the normality hypothesis. An ANOVA or Kruskal–Wallis test was performed to analyze data from more than 3 groups. Assessment of survival outcome was conducted using Kaplan–Meier survival analysis curves and the log-rank test was used to compare the groups. [SAS/STAT] software version 9.4 (SAS Institute Inc., Cary, NC, USA) and R foundation were used for data analysis and statistical computing (Vienna, Austria), accordingly [32]. 

## 3. Results

### 3.1. Patient Characteristics

This study comprised 183 CML patients. The overall mean age of all patients was 34.6 years. However, the mean age for CP-CML and AP-CML patients was 33.5 and 35.6 years, respectively. Regarding gender, CML was more common in males, as they constituted 60.5% of the total, while females made up only 39.5%, giving a male-to-female ratio of 1.6:1 (*p* = 0.02). Moreover, the male-to-female ratios for CP-CML and AP-CML patients were 1.5:1 and 2:1, respectively. The mean hemoglobin at diagnosis was 10.1 g/dl, the mean white blood cell count was 317.9 × 10^9^/L, and the mean platelet count was 400.2 × 10^9^/L. Furthermore, anemia and leukocytosis of more than 50 × 10^9^/L were observed in more than two-thirds of the patients at diagnosis. In addition, various types of treatment were given to patients, including TKIs and chemotherapy. Overall, characteristics including hemoglobin level, WBC count, platelet count, treatment type, hepatomegaly, splenomegaly, and survival status were significantly altered in AP-CML patients compared to CP-CML patients. However, as these clinical parameters cannot serve the purpose of early detection of patients at risk of CML progression, genetic analysis of the cases (AP-CML) and controls (CP-CML) was carried out using NGS. The comparison between CML phases regarding patients’ demographic and laboratory characteristics is displayed in Table 1.

### 3.2. Next-Generation Sequencing (NGS)

As per the objective of the study to find a common biomarker exclusively specific to AP-CML, NGS-based exome sequencing showed that the FANCD2 gene was mutated in all but one (59/60 = 98.3%) of the AP-CML patients but in none of the CP-CML patients or healthy controls (Supplementary Table S1: FANCD2 IGV). A novel splice-site mutation was detected at genomic position 10,106,408 on chromosome 3p25.3 leading to cytosine to thymine substitution (c. 2022-5C>T) in the FANCD2 gene. This gene is an important member of FA pathway genes. As this mutation was shared by a majority of (98.3%) AP-CML patients but not CP-CML patients or any of the healthy controls from genomic databases, it suggests the mutated FANCD2 gene’s association with early disease progression in CML. Access to data generated by next-generation sequencing can be obtained from NCBI, to which it was submitted, at (https://www.ncbi.nlm.nih.gov/bioproject/PRJNA1119181, accessed on 3 March 2024).

### 3.3. Validation of Mutation by Sanger Sequencing

**Figure 1:**
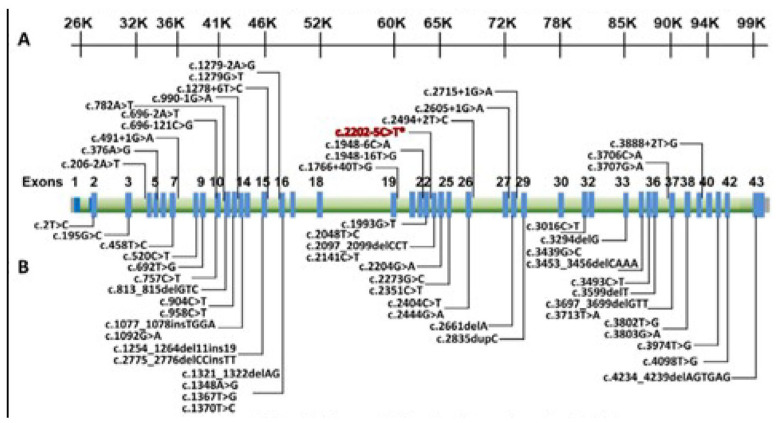
(**A**) FANCD2 locus at chromosome 3: 10,026,414–100,99,660 bp using UCSC genome browser GRCh38/hg38. (**B**) Exon–intron structure of FANCD2 gene in which exons are shown as numbers from 1 to 43. Splice-site location of intron 22 harboring mutation c.2022-5C>T is red-highlighted with asterisk symbol (*).

**Figure 2:**
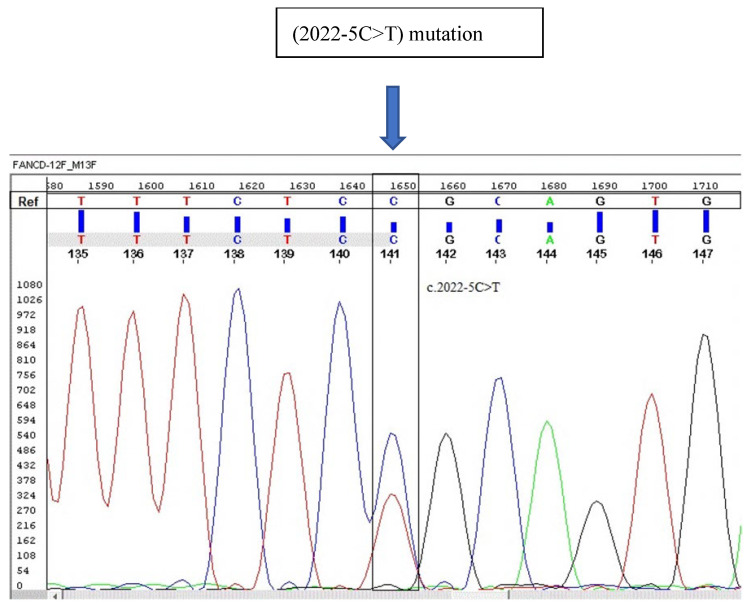
Electropherogram showing cytosine to thymine FANCD2 mutation at gene position 2022 (2022-5C>T) that is a splice site of intron 22, as detected by next-generation sequencing and confirmed by Sanger.

**Figure 3:**
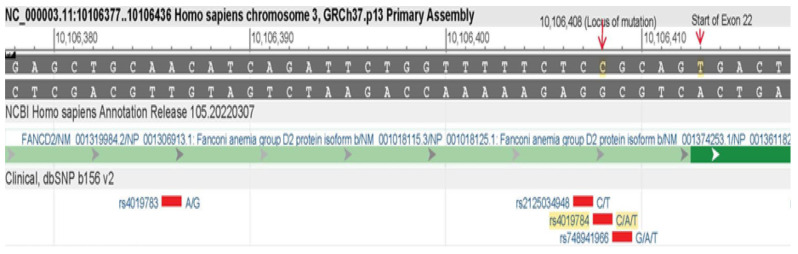
Genomic mapping of FANCD2 showing its position just 5 nucleotides downstream of exon 22, making it a splice-junction mutation.

A heterozygous variant (C2022T) was found and validated by Sanger sequencing. The FANCD2, c. 2022-5C>T (genomic position 10,106,408) detected by NGS was confirmed by Sanger sequencing (Figure 1 and Figure 2). Genomic mapping of this mutant shows that it is located on the intronic–exonic boundary, just five nucleotides upstream of exon 22 of FANCD2 (Figure 3). The GnomAD browser shows that this variant falls into the splice region between intron 22 and exon 22 of the FANCD2 gene and has a very low allele frequency of 0.0005100 in the general population (https://gnomad.broadinstitute.org/variant/3-10106408-C-T?dataset=gnomad_r2_1, accessed on 3 March 2024). The sequence data file generated from the VCF files shows that the VAF of FANCD2 (c. 2022-5C>T) is 22/42, indicating its heterozygosity (Supplementary Table S1; https://doi.org/10.6084/m9.figshare.25957204.v2, accessed on 3 March 2024).

The findings of our work suggest that the FANCD2 (c. 2022-5C>T) mutation is a strong indicator and potentially very specific biomarker of the early progression of disease in CML patients that can help in the early identification of a subset of CML patients at risk of CML progression and thus can help make timely therapeutic decisions to delay or even avoid BC-CML using standard TKI-based therapeutic regimes.

## 4. Discussion

We carried out this study to validate FANCD2 gene mutation in accelerated phase (AP) CML. We found that the FANCD2 gene was mutated in all patients in the AP compared to healthy individuals and chronic-phase (CP) CML patients. Although FANCD2’s genomic stability is assumed to be mostly regulated by FANCD2, monoubiquitination of FANCD2 in the cell activates the FANCI/FANCD2 complex formation, which recruits DNA repair proteins for interstrand crosslink (ICL) repair and replication fork protection, preventing DNA damage by mending the damaged DNA [33,34]. Defects and mutations in these proteins, both structural and conformational, can cause R-loop accumulation, contributing to genomic instability that is associated with many cancers [34]. FANCD-2 already has an established role in CML leukemogenesis [35]. It has been reported that FANCD2 plays a dual role in CML progression, with overexpression promoting cell survival and propagation, and inhibition of FANCD2 foci by the BCR-ABL fusion gene leading to genome instability [35,36]. Moreover, FANCD2 overexpression has been reported in leukemia drug resistance [37]. It has also been shown that FANCD2 downregulation and FANCD2-Ub inhibition lowered CD34+ CML cells’ clonogenic capacity and delayed BCR-ABL1 leukemogenesis in CML mouse models [36]. Moreover, it is already well documented that FANCD2 is involved in regulating homologous recombination (HR) repair [38]. Very recently, it has been reported that overexpression of the BCR-ABL fusion oncogene and other tyrosine kinases in leukemia leads to overexpression of DNA polymerase theta (DNA Pol θ) and accumulation of DNA–protein crosslinks (DPCs) containing DNA double-strand breaks by POLθ-mediated end-joining; inhibition of this polymerase can help eradicate BCR-ABL-positive stem cells [39,40]. This indicates the scientific and clinical benefit of studying FANCD2 and its related genes and associated mechanisms in leukemia for improving the diagnosis, prognosis, and therapeutics of leukemias. Nevertheless, the majority of documented evidence on FANCD2 mutations in CML is based on cell lines, and the evidence of their relationship with CML development is limited. Our previous work reported the FANCD2 mutant’s (c. 2022-5C>T) association with BC-CML, which needed its reproducibility validated as well as clinical validation of this important gene mutation as a biomarker for early disease progression in CML [20].

Chronic myeloid leukemia (CML) has three stages, namely the chronic phase (CP), the accelerated phase (AP), and the blast crisis (BC) [41]. Tyrosine kinase inhibitors (TKIs) have revolutionized the treatment of CML, with overall survival of CP-CML comparable to the general population, yet TKIs work well mainly in CP-CML and to some extent in AP-CML, and are less effective in BC-CML, with an average overall survival of less than a year [1,12]. Therefore, the early recognition of CML patients at risk of disease progression can help in timely interventions with appropriate TKIs or other therapeutic modalities to delay or avoid disease progression. On the other hand, although some markers of disease progression like ABL kinase domain mutations are available, no universal and exclusively specific molecular biomarkers exist to diagnose CML patients during early disease progression [14].

Our studies found that the mutant FANCD2 was exclusively associated with 100% of BC-CML cases [20]. Therefore, the current study was designed to detect the FANCD2 mutation in AP-CML (early progression phase) patients and to investigate and clinically validate its potential as a novel biomarker of early CML progression from CP to AP. Our study comprised 123 CP-CML (control group) and 60 AP-CML patients. Mean hemoglobin level, WBC count, platelet count, treatment type, hepatomegaly, splenomegaly, and survival status of AP-CML patients were significantly different from CP-CML patients. However, because these clinical parameters are not capable of serving the objective of early diagnosis of individuals at risk of CML progression, the next-generation sequencing (NGS) technique was utilized to conduct a genetic analysis on both the cases (AP-CML) and the controls (CP-CML).

Earlier studies of our group had discovered the FANCD2 mutant 2022-5C>T in every single BC-CML patient, and by employing whole-exome sequencing, they had narrowed their search for a “Common and specific biomarker for BC-CML” to only those genes that were mutated in all patients with BC-CML and not in any patients with CP-CML or healthy controls [20]. In the present study, we applied the same approach to narrow down the list of genes that were mutated in all or the majority of cases (at least 90%) of AP-CML or BC-CML. Furthermore, it was necessary to replicate the previous study, and additional clinical validation of the study was required to be carried out with a larger number of patients who were diagnosed with CML.

In our recent studies, the NGS performed for this investigation revealed that the FANCD2 gene was mutated in all but one of the AP-CML patients (59 out of 60, or 98.3%). As a result, the FANCD2 gene was investigated for subsequent clinical validation studies. Our NGS analysis confirmed our previously reported unique splice-site mutation in the FANCD2 gene (c.2022-5C>T). This mutation was detected in the majority (98.3%) of AP-CML patients but in none of the CP-CML patients or healthy control sequences from genomic databases. The mutation was confirmed by Sanger sequencing. FANCD2 is a member of the Fanconi anemia pathway genes involved in DNA repair and genomic stability, and aberrations of this gene are associated with many cancers.

In our study, the splice-site mutation between intron 22 and exon 23 resulted in the intron 22 variant. The function of the intron 22 variant on FANCD2 monoubiquitination remains uncertain, but it could be predicted since it is close to intron 19, which is the FANCD2 monoubiquitination site [42]. A study conducted in China identified a missense mutation c.3713T>A; p.M1238K in the FANCD2 gene that leads to non-expression of the FANCD2 protein. Moreover, function studies were carried out to show that other splice-site mutations in the FA gene cause exon skipping [43]. Another study reported 25 intronic variants and 6 silent coding variants that lead to familial breast cancer, one of which was in exon 23 c.2148 C>G, resulting in a T716T protein change [42].

The FANCI-FANCD2 heterodimer is present where the FA pathway comes together [43]. It serves as a substrate for the FA core complex as well as a potential collecting site for proteins involved in downstream DNA repairs, such as FAN1 nuclease and other FANC proteins [44]. Despite only possessing a 14% conservation in their solenoidal structures, FANCI and FANCD2 are known to have striking similarities in the crystal lattice of mouse FANCI-FANCD2 [33]. More than 97% of FA patients have a deficiency caused by mutations in the genes encoding FANCD2 and FANCI [45]. At the region of DNA damage, the FA proteins function as a ubiquitin E3 ligase to monoubiquitinate the FANCI-FANCD2 pair [46]. This results in enlisting downstream nucleases with ubiquitin-binding domains to restore the interstrand DNA bridge [47]. Although monoubiquitination and FA pathway activation necessitate DNA binding of FANCI-FANCD2, it is not certain how it triggers the DNA repair [48,49]. 

High FA gene expression is typically associated with chemoresistance; the high level of FANCD2 expression is associated with reduced chemotherapy sensitivity and a higher tumor mutation rate. It has been observed and reported in breast, lung, and ovarian cancers, thus resulting in reduced survival time [50,51]. FA pathway inhibition by targeted therapies is a promising approach for improving the efficacy of chemotherapy due to its role in chemoresistance across a wide range of cancers [52]. In early studies, curcumin, wortmannin, H-9, and alsterpaullone were found to inhibit FANCD2 by apoptosis through the NFκB pathway [53]. A study further assessed monoketone analogues of curcumin and found that EF24 was more specific and active against the monoubiquitination of FANCD2 [54]. Lastly, a recent study has identified CU2 as a compound that shows potential biochemical ubiquitylation selectivity and activity against the FA pathway [55]. Currently, three PARP inhibitors that target the FA pathway, olaparib, rucaparib, and niraparib, are FDA approved for treating relapsed breast and ovarian cancer [56].

Furthermore, there have been other reports that linked cancer incidence with FA pathway mutations [54]. The previously mentioned mutations were reported to cause bone marrow failure in FA. Also, the well-known genes BRCA1 and BRCA2 that predispose to breast cancer are considered to be part of the FANC gene family. This system is frequently referred to as the FA-BRCA pathway given the growing relationship between FA and the genes for breast cancer [57]. In addition, two studies have investigated the relationship between FANCD2 and breast cancer. In a Chinese study, poor prognosis was observed in sporadic breast cancer patients with high levels of FANCD2 [58]. A study conducted in Finland supports these findings by showing a significant association of variant (c.2715 + 1G > A) in the FANCD2 gene with breast cancer [59]. Also, studies carried out on ovarian carcinoma samples verified that the risk of recurrence and death are highly associated with the expression level of FANCD2 [38,60]. In contrast, a study conducted on 181 ovarian cancer patients provided evidence of an increased survival rate in patients with the FANCD2 mutation [61]. On the basis of its upregulation, FANCD2 may contribute to tumorigenesis and impart an unfavorable prognosis to various types of cancers, including esophageal squamous cell carcinoma, head and neck cancer, nasopharyngeal carcinoma, and lung adenocarcinoma [62,63,64,65].

In our study, the impact of the FANCD2 mutation on CML progression and drug resistance highlights the relationship between FANCD2 and the progression of CML to the advanced phase [17,35].

## 5. Conclusions

We clinically validated that the FANCD2 gene was mutated (c. 2022-5C>T) in all but one (98.3%) of the AP-CML patients but in none of the CP-CML patients or healthy controls. FANCD2 is a member of the Fanconi anemia pathway genes involved in DNA repair and genomic stability. Therefore, our study reports the FANCD2 (c. 2022-5C>T) mutation as a new molecular marker for early CML progression. We recommend prospective clinical studies to further validate the role of this biomarker.

## Figures and Tables

**Table 1:** A comparison between demographic and laboratory characteristics of CP- and AP-CML patients included in this study.

Characteristics	Patient Groups (%) *n*		
	CP-CML	AP-CML	*p* Value
	(67.2) 123	(32.8) 60	
**Mean age (Range)**	33.5 (range 7–69)	35.6 (range = 27–43)	
**Gender**			
Male	(60.2) 74	(66.67) 40	0.60
Female	(39.8) 49	(33.33) 20	0.59
**Male: Female Ratio**	1.5:1	2:1	0.02
**Mean Hemoglobin (g/dL)**	10.1		
**Mean WBC count (×10^9^/L)**	313.7	315	
<50	(16.3) 20	(20) 10	0.82
>/=50	(83.7) 103	(80) 50	0.02
*p*-value	0.005	0.02752	
**Platelets (×10^9^/L) Mean**	400.2		
<450	75 (61)	40 (66.7)	
>/=450	33 (26.8)	20 (33.3)	
No data found	15 (12.2)	0	
***p*-value**	0.0011	0.47	
**Imatinib**			
Yes	(66.7) 82	(66.7) 40	0.72
**Interferon**			
Yes	(33.3) 41	(0) 0	0.0038
**Chemotherapy**			
Yes	(8.1) 10	(66.7) 40	<0.0001
**Splenomegaly**			
<5 cm	(3.3) 4	(0) 0	0.43
5–8 cm	(7.3) 9	(16.7) 10	0.061
>8 cm	(56.9) 70	(83.3) 50	0.07
**No splenomegaly**	(32.5) 40	(0) 0	0.004
**Hepatomegaly**			
Yes	(28.5) 35	(66.7) 40	0.001
**Survival Status**	(100) 123	59 (98.3)	0.0003
Confirmed deaths	0	1 (1.7)	0.0003
Frequency of FANCD2 (c. 2022-5C>T)	0 (00)	59 (98.3)	0.0003

## Data Availability

Access to data made by next-generation sequencing can be obtained from NCBI, to which it was submitted, at https://www.ncbi.nlm.nih.gov/bioproject/PRJNA1119181. Supplementary Data related to the NGS of FANCD2 was submitted to an online data server (Supplementary Table S1; https://doi.org/10.6084/m9.figshare.25957204.v2, accessed on 3 March 2024). Validation of the mutation was performed by Sanger Sequencing.

## Supplementary Materials

The following supporting information can be downloaded at: https://www.mdpi.com/article/10.3390/hematolrep16030045/s1, Supplementary Table S1; https://doi.org/10.6084/m9.figshare.25957204.v2, accessed on 3 March 2024).
