# Supplementary material for: Clinical Validation of the Somatic FANCD2 Mutation (c.2022-5C>T) as a Novel Molecular Biomarker for Early Disease Progression in Chronic Myeloid Leukemia: A Case–Control Study
Source: Hematol Rep. 2024 Jul 8;16(3):465–78. doi: 10.3390/hematolrep16030045 (PMC11270283; doi:10.3390/hematolrep16030045)
Supplement: Supplementary file 1 [file hematolrep-16-00045-s001.zip › hematolrep-2968304-supplementary.pdf]

#CHROM	POS	REF	ALT	DP	AD	QUAL	MQ	Zygosity	FILTER	Effect	Putative_Impact	Gene_Name	Feature_Type	Feature_ID	Transcript_BioType	Rank/Total	HGVS.c
chr3	10,106,408	C	T	71	8	51.77	57.04	HET	MG_SNP_Filter	splice_region_variant&intron_variant	LOW	FANCD2	transcript	NM_033084.3	Coding	22/42	c.2022-5C>T

Supplementary Table 1: FANCD2 VAF data from next-generation sequencing studies
